# Defining a Multi-Omic, AI-Enabled Stool Screening Paradigm for Colorectal Cancer: A Consensus Framework for Clinical Translation

**DOI:** 10.3390/cancers18060909

**Published:** 2026-03-11

**Authors:** Arturo Loaiza-Bonilla, Yan Leyfman, Viviana Cortiana, Rhys Crawford, Shivani Modi

**Affiliations:** 1 Division of Hematology and Oncology, Department of Medicine, St. Luke’s Cancer Center, St. Luke’s University Health Network, Bethlehem, PA 18015, USA; 2 Massive Bio, Boca Raton, FL 33487, USA; 3 Meyer Cancer Center, New York Presbyterian Hospital, New York, NY 10021, USA; yan.leyfman@nyp.org; 4 University of Bologna, 40126 Bologna, Italy; vivycort02@gmail.com; 5 Tulane University of Louisiana, New Orleans, LA 70118, USA; rcrawfordus@gmail.com; 6 Thomas Jefferson University, Philadelphia, PA 19107, USA; shivani.modi@jefferson.edu

**Keywords:** colorectal cancer screening, gut microbiome, multitarget stool DNA, host DNA methylation, artificial intelligence, multi-omic biomarkers, diagnostic accuracy reporting, cancer prevention

## Abstract

Colorectal cancer (CRC) develops through both conventional adenoma–carcinoma and serrated neoplasia pathways, yet noninvasive screening still under-detects the advanced precursor lesions that enable true cancer prevention. Stool-based screening reduces CRC mortality, but its preventive impact remains constrained by limited detection of advanced precancerous lesions (APLs), including advanced adenomas and sessile serrated lesions. Next-generation multitarget stool DNA assays (mt-sDNA; e.g., Cologuard Plus) have established high sensitivity for CRC and specificity approaching 94%, leaving improved APL detection as the principal opportunity for innovation. This review presents a consensus framework for a multi-omic stool screening paradigm that integrates host epigenetic markers (DNA methylation) with gut microbiome features using artificial intelligence (AI). Multi-omics capture complementary layers of early tumor biology: epithelial shedding and field effects reflected in host methylation signals together with luminal ecological and inflammatory changes represented by microbial features. Evidence from cross-cohort microbiome studies indicates that microbial signatures provide an additive—rather than standalone—axis of information for CRC and its precursor lesions. Because microbiome-based models are highly susceptible to batch effects arising from collection devices, extraction chemistry, sequencing platforms, and bioinformatic pipelines, practical mitigation strategies are outlined, including harmonized pre-analytics, batch-aware study design, leakage-resistant validation, and computational harmonization. A translational roadmap linking analytical validity, locked-model development, and prospective colonoscopy-verified clinical validation is proposed, aligned with TRIPOD + AI, STARD, PROBAST-AI, SPIRIT-AI, CONSORT-AI, and DECIDE-AI reporting standards. Scenario modeling using BLUE-C prevalence estimates suggests that improving APL sensitivity from approximately 43% to 55–65% at ~94% specificity could translate to detecting roughly 13–23 additional advanced precancerous lesions per 1000 individuals screened, highlighting the potential prevention impact of a multi-omic approach. This framework aims to guide developers and clinical investigators toward next-generation stool tests capable of materially improving precursor-lesion detection while maintaining clinically acceptable specificity.

## Simple Summary

Most colorectal cancers can be prevented when advanced precancerous lesions are found and removed, yet many people do not undergo colonoscopy. Home stool-based tests help improve access, but current stool DNA tests still miss many advanced precancerous lesions (advanced adenomas and serrated precursors). This review explains why combining two complementary signals from the same stool sample may help: host DNA methylation markers shed from the colon lining and patterns in the gut microbiome. Artificial intelligence (AI) can fuse these signals into a single score and provide clinician-friendly explanations. Because microbiome data are sensitive to collection and laboratory differences, practical steps are outlined to standardize pre-analytics and reduce batch effects. A stepwise evidence-generation roadmap aligned with modern reporting standards is also presented to support real-world clinical translation.

## 1. The Evolving Landscape of Noninvasive Colorectal Cancer Screening

### 1.1. Clinical Imperatives: Adherence Gaps and the Precancerous Lesion Detection Deficit

Colorectal cancer represents a significant global health burden, ranking as the second most common cause of cancer death in the United States [1]. Early detection is paramount, as five-year survival rates exceed 90% for localized disease but decline precipitously with metastasis. While colonoscopy is the gold standard for both detection and prevention through polypectomy, its invasive nature, cost, and resource demands contribute to persistent gaps in screening adherence. Despite multiple endorsed modalities, over 20 million U.S. adults are not up to date with colorectal cancer screening, according to national surveillance estimates (e.g., ACS reports) [2]. Company communications sometimes cite higher figures (48 million), but these vary by data source, denominator, and definition of ‘unscreened’ [3]. Noninvasive stool-based tests, such as the Fecal Immunochemical Test (FIT) and mt-sDNA assays, have emerged as critical tools to bridge this adherence gap by offering convenient, at-home options [4]. However, the ultimate clinical and economic value of any screening program lies not just in detecting extant cancers but in preventing them entirely by identifying and enabling the removal of precancerous lesions, primarily advanced adenomas and sessile serrated lesions [5]. It is in this preventative capacity that current noninvasive modalities exhibit their most significant clinical deficit. FIT, for example, has low sensitivity for advanced adenomas, and while mt-sDNA tests have improved upon this, a considerable detection gap persists, representing the key unmet need in colorectal cancer prevention [4].

**Figure 1:**
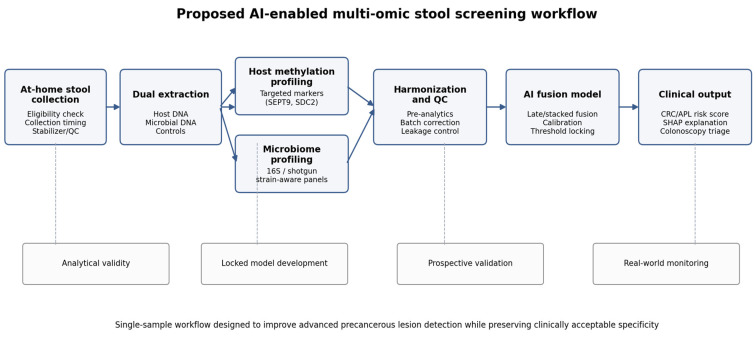
Proposed AI-enabled multi-omic stool screening workflow showing harmonized collection, dual extraction, host-methylation and microbiome profiling, bias-aware fusion modeling, explainable risk reporting, and colonoscopy-directed clinical action.

Figure 1 summarizes the proposed workflow.

### 1.2. The Performance Benchmark: Multitarget Stool DNA (Mt-sDNA) and the Next-Generation Cologuard Plus

The commercial and clinical landscape of noninvasive CRC screening is dominated by multitarget stool DNA (mt-sDNA) testing. The first-generation assay (Cologuard^®^) combines fecal hemoglobin detection with selected host DNA methylation and mutation markers shed from the colonic epithelium. Its successor, Cologuard Plus (next-generation mt-sDNA), launched in 2025, has reset the performance benchmark against which new noninvasive screening technologies must be measured [3].

In the pivotal >20,000-participant BLUE-C study, the “BLUE-C values” used throughout this manuscript refer to the peer-reviewed per-participant diagnostic accuracy estimates reported for next-generation mt-sDNA in an average-risk screening cohort [5]. Cologuard Plus achieved 93.9% sensitivity for CRC [5]. The reported specificity range of 90.6–92.7% reflects differences in how the reference-negative colonoscopy group was defined, particularly whether minor or nonadvanced findings were classified within or outside the true-negative population [5]. FDA labeling applies an average-risk subgroup analysis and a stricter negative definition, yielding an operating point of 95% CRC sensitivity at approximately 94% specificity [6,7]. In this manuscript, BLUE-C values are used when describing peer-reviewed trial performance with empirical data noted in Table 1, whereas the FDA-labeled operating point is used for illustrative fixed-specificity yield modeling in Table 2.

This represents a notable improvement over first-generation mt-sDNA, which reported approximately 92% CRC sensitivity and 87–90% specificity [8]. Cologuard Plus also improved detection of advanced precancerous lesions (APLs), with 43.4% sensitivity for APLs overall and approximately 74% sensitivity for lesions with high-grade dysplasia in BLUE-C [5]. These performance gains are important because they improve referral efficiency while maintaining a strong prevention-oriented signal for the highest-risk precursor lesions.

Compared with the original mt-sDNA assay, the next-generation test demonstrated an approximately 30–40% relative reduction in false positives, corresponding to ~6–9% versus ~10–13% in BLUE-C (depending on the negative definition) [5]. This range is consistent with the FDA Summary of Safety and Effectiveness Data and company-reported summaries describing reductions of >30% to nearly 40% [6,7]. Beyond numerical performance, Cologuard Plus illustrates several clinically relevant strengths for the next phase of stool screening: preservation of an at-home single-sample workflow, compatibility with existing mt-sDNA logistics, fewer unnecessary follow-up colonoscopies than legacy mt-sDNA, and stronger enrichment for high-grade dysplasia-containing lesions [5,6,7].

Guardant Shield (blood-based): The Shield cell-free DNA (cfDNA) assay demonstrated sensitivity for colorectal cancer (CRC) of 83.1% with specificity for advanced neoplasia of 89.6% in the ECLIPSE clinical study, but sensitivity for advanced precancerous lesions (APL) was 13.2%, underscoring the current gap in preventive lesion detection relative to stool-based multitarget stool DNA modalities [9].

### 1.3. The Unmet Need: Quantifying the Opportunity for Improvement

The performance of Cologuard Plus has created a “benchmark squeeze” in the diagnostics market. Its high specificity and CRC sensitivity have raised the barrier to entry, making it insufficient for a new test to compete solely on the basis of being noninvasive. Any new entrant must now demonstrate clear superiority on a key clinical metric. The most significant remaining vulnerability, and thus the greatest opportunity for innovation, is the persistent gap in APL detection (approximately 43% APL sensitivity in BLUE-C), meaning that more than half of these critical precursor lesions are still missed by the leading stool-based test [5,6].

This reality has catalyzed a strategic pivot in the field, moving the narrative from “early cancer detection” to “proactive cancer prevention.” The emphasis on improving APL detection in Cologuard Plus underscores a market-wide recognition that the primary value proposition of screening is cancer prevention through the removal of precursor lesions. Consequently, the central goal for a next-generation multi-omic stool test is clear: to materially improve APL sensitivity beyond the current benchmark while preserving high specificity (approximately 94%) and CRC sensitivity comparable to next-generation mt-sDNA [5,6,7]. This is the clinical and translational rationale for integrating gut microbiome signals with host methylation markers. This conceptual shift is illustrated in Supplementary Figure S7.

For scenario modeling and trial-design discussion, an APL sensitivity range of 55–65% at approximately 94% specificity is used as a pragmatic target window. At BLUE-C-like prevalences (CRC = 4.86/1000; APL = 106.26/1000) [5], improving APL sensitivity from ~43% to 55–65% would correspond to detecting roughly 13–23 additional APLs per 1000 screened (Table 2), a magnitude likely to be clinically meaningful while keeping follow-up colonoscopy volumes within a potentially manageable range. More broadly, multi-omic cancer research is valuable because early neoplasia is expressed across multiple biological layers rather than a single analyte; in a stool assay, host methylation may capture epithelial shedding and field effects, whereas microbial features may capture ecological and inflammatory perturbations that are missed by hemoglobin-dominant tests. Importantly, this 55–65% range is an aspirational modeling target rather than an empirically established requirement, and it should be refined by prospective screening studies and payer-aligned health-economic analyses [10]. Empirical performance across currently available or publicly described stool- and blood-based options is summarized in Table 1.

**Table 1:** Empirical performance characteristics of leading and emerging colorectal cancer screening modalities.

Modality	Sample/Interval	Sens. (CRC)	Sens. (APL/HGD)	Specificity	Key Advantages and Limitations
Colonoscopy	NA/10 years	>95%	High	High	Reference standard; diagnostic and therapeutic. Invasive; prep/sedation; resource limits [4].
FIT	Stool/1 year	67.3%	23.3%	94.8–95.7%	Low cost and scalable. Low APL sensitivity; annual adherence required [5].
Cologuard (original mt-sDNA)	Stool/3 years	92.3%	42.4% (HGD 69.2%)	86.6%	Higher CRC/APL sensitivity than FIT. Lower specificity; more follow-up colonoscopies [8].
Cologuard Plus (next-gen mt-sDNA)	Stool/3 years	93.9%	43.4% (HGD ~74%)	90.6–92.7%	Higher specificity than legacy mt-sDNA; strong HGD detection. More than half of APLs remain missed [5,6,7].
Shield (Guardant; cfDNA blood)	Blood/not established	83.1%	13.2%	89.6%	Clinic-based blood draw; limited APL detection [9].
Viome (stool RNA/microbiome)	Stool/not established	Not public	Not public	Not public	Development-stage stool RNA/microbiome platform; average-risk prospective data remain limited [11,12,13].
BiotaX Labs (stool microbiome)	Stool/not established	Not public	Not public	Not public	Development-stage stool microbiome test; prospective colonoscopy-verified screening validation is still needed [14].
Freenome (blood-based multiomic)	Blood/not established	79.2%	12.5%	91.5%	Blood-based multiomic approach; limited APL detection in published validation [15,16].

Abbreviations: CRC, colorectal cancer; APL, advanced precancerous lesion; HGD, high-grade dysplasia. For development-stage platforms, public evidence is limited and intended-use claims remain provisional until prospective average-risk screening validation with colonoscopy as the reference standard.

To avoid conflating empirical test performance with projections, Table 1 is limited to published performance metrics, whereas Table 2 reports only modeled per-1000 yield scenarios derived from those published inputs. The goal is not to claim observed performance for a new assay, but to show the magnitude of advanced precancer detection gain that would likely be required to change clinical utility. Downstream clinical impact was modeled per 1000 average-risk adults screened using prevalences observed in the BLUE-C trial and published test performance estimates from BLUE-C (FIT and next-generation mt-sDNA) [5], the original DeeP-C legacy mt-sDNA study [8], and FDA/company materials for Cologuard Plus [6,7]. For comparability, modeled scenarios use a fixed specificity of 94% (the legacy mt-sDNA row is shown at its published operating point, approximately 86.6%) [8]. Under these assumptions, FIT would detect ~3.3 CRCs and ~24.8 APLs per 1000 screened, whereas Cologuard Plus (95% CRC sensitivity at ~94% specificity) increases APL detection to ~46.1 per 1000 with a commensurate rise in follow-up colonoscopies [6,7]. A proposed multi-omic test that maintains CRC sensitivity near 95% and achieves APL sensitivity in the 55–65% range would detect roughly 58–69 APLs per 1000 (with follow-up colonoscopies of ~116–127 per 1000), representing a substantial incremental precancer detection yield versus FIT and Cologuard Plus while generating a moderate increase in colonoscopy referrals. Calculation details and assumptions are provided in the Table 2 legend and scenario note.

**Table 2:** Scenario analysis only: modeled per-1000 yields for published comparator inputs and hypothetical multi-omic operating points (not empirical assay results).

Test (Operating Point)	CRC Sens (%)	APL Sens (%)	Spec (%) Used	Detected CRCs/1000	Detected APLs/1000	Follow-Up Colonoscopies/1000 (Positives)
Published comparator scenario—FIT (BLUE-C input) [5]	67.3	23.3	94.0 (modeled)	3.3	24.8	81.4
Published comparator scenario—legacy mt-sDNA (DeeP-C input) [8]	92.3	42.4	86.6 (published)	4.5	45.1	168.6
Published comparator scenario—Cologuard Plus (FDA-labeled operating point input) [6,7]	95.0	43.4	94.0	4.6	46.1	104.1
Hypothetical multi-omic target—APL 55% (assumed CRC sens 95%, spec 94%)	95.0	55.0	94.0	4.6	58.4	116.4
Hypothetical multi-omic target—APL 60% (assumed CRC sens 95%, spec 94%)	95.0	60.0	94.0	4.6	63.8	121.7
Hypothetical multi-omic target—APL 65% (assumed CRC sens 95%, spec 94%)	95.0	65.0	94.0	4.6	69.1	127.0

Scenario note: Every value in Table 2 is a model output generated from published sensitivity, specificity, and prevalence inputs; none represents observed per-1000 outcomes from a prospective trial. The rows labeled “Hypothetical multi-omic target” are theoretical operating points intended to guide assay design and trial planning. Clinical impact is modeled per 1000 average-risk adults screened using BLUE-C prevalences (CRC = 4.86/1000; APL = 106.26/1000). Values show detected CRCs and APLs and the number of follow-up colonoscopies (positive screens) for FIT, legacy mt-sDNA (published operating point), Cologuard Plus (modeled at the FDA-labeled operating point), and candidate multi-omic target windows. Assumptions: 94% specificity for all modeled scenarios except legacy mt-sDNA (published specificity ~86.6%); CRC sensitivity for the proposed multi-omic test was assumed to be 95%.

Pre-analytic factors are a major and under-recognized source of heterogeneity in reported stool-based colorectal screening test performance and can materially bias estimates of sensitivity, specificity, and downstream clinical impact. Across studies and manufacturer SOPs the most commonly applied exclusions and sample-level flags include recent systemic antibiotics (typical washout ~30 days), visible gastrointestinal bleeding (~14 days), colonoscopy or bowel preparation within a week of collection, recent rectal manipulation (48–72 h), shipping time/temperature excursions outside validated stability envelopes, low biomass/internal control failure, and evidence of laboratory or collection contamination. These pre-analytic events may produce false-negative (degraded or low target signal) or false-positive (blood or contamination) results and differentially affect assays that rely on host DNA, microbial signatures, or methylation markers. For comparative evaluations and meta-analyses, investigators should therefore (1) define and report all pre-analytic exclusion criteria a priori and the number of samples excluded for each reason, (2) report the internal control metrics and thresholds used to define low-biomass failures, (3) document time-temperature monitoring and the proportion of shipments with excursions, and (4) adopt a standardized mitigation algorithm (intake QC, reserve aliquots for repeat testing, batch quarantine and re-testing for contaminated runs, and explicit re-collection windows). Harmonized reporting using these elements (analogous to STARD/PRISMA expectations for diagnostic accuracy and systematic reviews) will improve interpretability across studies, reduce selection bias in primary analyses, and enable more accurate modeling of clinical and health-economic outcomes.

To ensure reproducibility and regulatory readiness, a “Minimum Translational Checklist” (Table 3) is proposed to synthesize key pre-analytical and validation requirements. Full operational details, including specific exclusion definitions and feature-handling protocols, are provided in Supplementary Tables S1 and S2 [17,18].

Preventing feature leakage is essential for valid, reproducible claims about diagnostic performance and downstream clinical impact [19]. In comparative evaluations of stool- or blood-based screening assays, an explicit a priori ban is recommended on any features that would not be available at the time of screening—most importantly hemoglobin immunoassay results obtained during diagnostic triage and any colonoscopy-derived variables (polyp histology, size, or pathology timestamps) [19]. All preprocessing steps (imputation, scaling, encoding, feature selection) must be fit exclusively within training partitions; supervised encodings based on label aggregates must never be computed on the full dataset. To produce conservative, deployment-relevant estimates, investigators should use nested cross-validation (inner loop for hyperparameter tuning and feature selection; outer loop for unbiased performance estimation) combined with GroupKFold-style partitioning so that all samples from the same clinical site and the same participant are contained entirely within a single fold [20,21]. Thresholds for clinically actionable operating points (e.g., specificity fixed at 94%) should be derived from training folds only and then applied to held-out data when reporting yield metrics (per-1000 screened), with 95% CIs from the outer folds [17].

Temporal robustness and site generalizability must be evaluated explicitly rather than assumed [22]. Chronological (time-forward) holdouts—training on earlier collections and testing on later collections—simulate prospective deployment and reveal calibration drift; when drift is observed, pre-specify recalibration or retraining strategies (for example, Platt scaling on a small temporal holdout or rolling retraining windows) and report their effect [18,22]. Finally, routine leakage diagnostics (label permutation, injection of known post-label features as positive controls, per-site performance tables, and metadata-feature correlation checks) should be performed and reported in every validation study [23]. Adopting these feature-handling and validation conventions in methods and Supplementary Materials will reduce optimistic bias, increase reproducibility, and improve the utility of results for guideline committees and payers who must judge real-world clinical and economic value (Supplementary Table S2) [17].

Explainability is critical for clinician acceptance of complex multi-omic screening tests because it translates a black-box probability into human-interpretable drivers that can be reviewed alongside clinical metadata [24]. Local attribution methods such as SHAP (SHapley Additive Explanations) quantify each feature’s contribution to an individual prediction and therefore enable patient-level explanations (waterfall plots, force plots) that show how microbiome taxa and host epigenetic markers jointly shift a screening probability. Practically, a concordant signal—for example, enrichment of CRC-associated taxa such as Fusobacterium nucleatum together with high SEPT9 or SDC2 methylation—provides stronger mechanistic plausibility for a positive call than a microbiome-only signal and thus increases clinician confidence; conversely, microbiome-only calls without host methylation support warrant careful review for confounders (bleeding, inflammation, recent antibiotics). SHAP has been widely adopted for tree-based models and provides consistent local attributions that can be aggregated to show feature importance and selection frequency across the cohort (Table 4) [25].

To operationalize explainability in validation and deployment, it is recommended that manuscripts or regulatory packages (1) include one representative patient-level SHAP panel per outcome class (CRC, APL, control) to illustrate concordant versus discordant signal patterns, (2) report the top taxa and methylation markers with median SHAP contributions and selection frequency across outer folds, and (3) provide a clinician-facing interpretation template (e.g., “Microbiome: Fusobacterium↑; Epigenetics: mSEPT9↑ -> high plausibility for neoplasia; recommend expedited diagnostic colonoscopy”). Such reporting documents biological alignment (e.g., Fusobacterium enrichment in CRC) and provides an audit trail for individual decisions; it also allows secondary review by pathologists and gastroenterologists when microbiome signatures suggest alternative diagnoses (inflammation, recent bleed) [26]. For methodology, the XAI workflow exemplified by Novielli et al. should be followed, with local attributions underpinned by robustness checks (permutation of labels, feature-ablation tests, and consistency across folds) to ensure that SHAP attributions are stable and clinically meaningful (Table 4) [24,25].

## 2. The Gut Microbiome: An Independent and Synergistic Axis for CRC Detection

### 2.1. Foundational Evidence: Cross-Cohort and Meta-Analytic Validation of Microbial Signatures

The rationale for incorporating microbiome analysis into CRC screening is built on a foundation of large-scale studies demonstrating that the gut microbiome harbors a robust, reproducible, and generalizable signal for colorectal neoplasia. This signal is not an artifact of a single study, methodology, or population. Multi-cohort analyses have consistently identified universal bacterial markers that distinguish CRC cases from healthy controls across geographically and ethnically diverse populations [27].

A landmark multi-cohort study by Dai et al. analyzed metagenomes from Chinese, European, and U.S. cohorts and identified universal bacterial markers, achieving an area under the receiver-operating-characteristic curve (AUROC) of approximately 0.80 for CRC classification [28]. Subsequent large meta-analyses of fecal metagenomes have reinforced that microbiome signatures are reproducible across populations, but also that apparent performance depends on cohort composition and technical processing choices [27].

Across these cross-cohort efforts, taxa repeatedly enriched in CRC include Fusobacterium nucleatum, Parvimonas micra, Peptostreptococcus stomatis, Clostridium symbiosum, Gemella morbillorum, and Bacteroides fragilis, among others [27,28]. When aggregated into multi-taxa microbial risk scores or machine-learning classifiers, external-cohort AUROCs are commonly reported in the ~0.62–0.82 range, highlighting both transferability of signal and the importance of rigorous harmonization, calibration, and prospective evaluation for clinical translation [27].

Cross-cohort model transfer analyses similarly suggest that microbiome-based risk scores can retain discriminative performance when trained in one cohort and evaluated in independent cohorts, although calibration drift and batch effects can degrade transportability. These observations motivate batch-aware study design (including site-stratified and time-forward splits), harmonized pre-analytics, and the use of dedicated correction frameworks (e.g., ComBat-seq, MMUPHin, and DEBIAS-M) as part of an external-validation strategy [29,30,31]. Importantly, the commonly reported cross-cohort AUROC range of about 0.62–0.82 is not sufficient for a stand-alone population screening assay; rather, it defines the likely ceiling of a microbiome-only approach in heterogeneous real-world cohorts and supports the central design choice of this framework: using the microbiome as an additive signal layered onto strong host markers, not as a replacement for mt-sDNA [27,29,30,31].

Mechanistic nuance and assay implications. Recent strain-resolved metagenomic work highlights important mechanistic and practical nuances that should guide assay design. In particular, strain-level variation within Fusobacterium nucleatum—including clade- and strain-specific virulence repertoires—has been linked to differential tumor tropism and functional interactions with the host epithelium [32]. These findings imply that a species-level hit on *F. nucleatum* may mask biologically relevant heterogeneity: some strains/clades are more strongly associated with tumorigenic processes than others. Moreover, emerging evidence points to host-microbe cross-talk at the epigenetic and epitranscriptomic level (for example, microbe-associated inflammation or metabolites driving promoter methylation or RNA-modification patterns in host epithelial cells), suggesting that concordant host methylation markers (e.g., SEPT9/SDC2) and microbial strain signals provide stronger mechanistic plausibility for neoplasia than either alone (see also Novielli et al. for an explainability workflow) [24].

Translationally, these mechanistic insights motivate moving beyond species-level detection toward strain-resolved targets in clinically deployed assays (for example, clade-specific qPCR or targeted amplicon assays that discriminate high-risk *F. nucleatum* lineages). Combining such strain-resolved microbial markers with host epigenetic readouts in a multi-omic panel both increases biological plausibility for positive calls and helps discriminate microbiome shifts due to confounders (bleeding, inflammation) from those more likely to reflect neoplasia. Including strain-level targets in marker panels also facilitates targeted validation and, where appropriate, the development of cost-effective molecular diagnostics suitable for large-scale screening.

### 2.2. Key Microbial Biomarkers: From Fusobacterium Nucleatum to Multi-Taxa Risk Scores

Research has converged on a set of key microbial biomarkers that are consistently enriched in the stool of individuals with CRC. These are often oral pathogens that are not typically dominant in a healthy colon, with Fusobacterium nucleatum, Parvimonas micra, and Peptostreptococcus stomatis being among the most frequently cited examples. Concurrently, a depletion of beneficial, butyrate-producing taxa is often observed. While early studies focused on the diagnostic potential of single markers like F. nucleatum, the field has rapidly evolved toward more sophisticated multi-taxa panels and machine learning-derived risk scores. These approaches provide more robust and accurate classification by capturing the complexity of the dysbiotic state associated with neoplasia. Notably, while taxa such as Fusobacterium nucleatum, Parvimonas micra, Peptostreptococcus stomatis, and Clostridium symbiosum have been observed across multiple cohorts, effect sizes and feature rankings vary by geography, population structure, sequencing depth, and analytical pipeline, underscoring the necessity of harmonization strategies and external, cross-laboratory validation [33].

The key translational question, however, is whether these microbial shifts are detectable early enough to improve precursor-lesion detection. Here the evidence is more modest but still encouraging. Systematic reviews indicate that microbiome-based classifiers usually discriminate CRC more strongly than adenomas, yet advanced adenomas already show partial enrichment of CRC-associated oral taxa and functional pathways, suggesting a biological continuum from control to adenoma to cancer [27,28,34]. Data for sessile serrated lesions are thinner and less standardized, so claims of screening-readiness would be premature. Taken together, current evidence supports microbiome use as an adjunct to improve APL sensitivity, especially for lesions that are less dependent on occult bleeding, but not yet as a sufficiently mature stand-alone test for precursor lesions.

### 2.3. Biological Plausibility and Clinical Synergy: Evidence for Combined Assays

The integration of host epigenetic and microbial markers is not merely a statistical exercise; converging evidence supports mechanistic links of biological interaction, with clinical studies suggesting that such combined markers can improve performance. The microbiome should not be viewed only as a correlated biomarker but as a potential upstream contributor to oncogenic processes. Evidence of biological cross-talk indicates that certain microbes can influence host epigenetic states. For instance, *F. nucleatum* has been associated with CRC-related DNA methylation patterns, providing suggestive evidence of interaction between microbial signals and host epigenetics detected by mt-sDNA tests [33]. While these links do not prove causation, mechanistic work shows that *F. nucleatum* can downregulate the m6A methyltransferase METTL3 via YAP-FOXD3 signaling, reducing m6A marks and promoting pro-metastatic programs in CRC—supporting a biologically plausible pathway from microbial exposure to host epigenetic regulation [35]. In practical terms, microbial signals may function as both risk-associated ecological features and disease markers: some taxa likely participate in carcinogenesis, whereas others simply track a permissive tumor microenvironment. This raises the possibility that a multi-omic test may capture both a contributing factor (the microbial community) and its downstream effect (host epigenetic changes), a combination that could enhance sensitivity for early-stage disease detection.

Proof-of-concept clinical data support the potential value of combining host and microbial markers, but the evidence base remains preliminary. In a 2023 case–control study, Fan et al. evaluated an mt-sDNA assay, serum carcinoembryonic antigen (CEA), and a small panel of fecal microbial genera in 54 CRC cases and 51 healthy controls; an integrated random-forest model achieved high apparent CRC sensitivity and specificity within this enriched design [36]. Because case–control studies with known cancers can overestimate performance relative to true average-risk screening populations, and because APL detection was not assessed, these results should be interpreted as hypothesis-generating rather than definitive clinical proof. Prospective screening studies with colonoscopy verification are needed to quantify incremental APL sensitivity and to confirm specificity at clinically relevant operating points. Separately, studies adding targeted microbial markers (for example, qPCR for *F. nucleatum*) to FIT have reported incremental gains for CRC and, in some cohorts, advanced adenomas, reinforcing the biologic complementarity of combined approaches [34].

From an assay-design perspective, absolute independence between modalities is not required. What matters is conditional complementarity—whether one modality adds information beyond the other at the same operating point in held-out data. Host methylation and microbiome features may be partially biologically linked, yet they arise from different compartments and time scales: epithelial shedding and field effects versus luminal ecology, inflammation, and microbial-host interaction. That partial correlation can therefore be advantageous: concordant signals increase biological plausibility, while discordant signals may flag confounding, recent inflammation, or earlier lesion biology. The key question is incremental value in external validation, not mathematical independence alone [24,34,35,36].

## 3. Artificial Intelligence as the Integration Engine for Multi-Omic Diagnostics

### 3.1. Machine Learning Architectures: From Ensemble Methods to Deep Learning

The translation of high-dimensional, multi-omic data into a single, clinically actionable result is a natural use case for AI and machine learning (ML). Microbiome features are compositional, sparse, and highly sensitive to batch effects, whereas methylation markers are often targeted and lower-dimensional; integrating the two therefore requires modality-aware preprocessing and validation. In practice, current stool-screening datasets are usually tabular, moderately sized, multi-source, and vulnerable to batch effects; for that reason, regularized logistic regression or elastic net, random forests, and gradient-boosted trees (e.g., XGBoost) are the most realistic primary model families for current development programs [37,38,39]. These methods handle nonlinear interactions, mixed feature scales, and moderate missingness while still permitting calibration analysis and local explanations.

Previously reported AI tools for stool-based CRC detection illustrate three useful archetypes. First, targeted feature-integration models, such as the random-forest approach of Fan et al., combine mt-sDNA-related markers, serum carcinoembryonic antigen, and selected fecal microbial genera in a compact classifier [36]. Second, large multi-cohort microbiome classifiers such as CRCpred use XGBoost to learn cross-study microbial patterns from metagenomic data [40]. Third, explainable stool-microbiome pipelines, exemplified by Novielli et al., pair classification with SHAP-based attribution so that the dominant taxa driving an individual prediction remain inspectable [24]. Collectively, these tools are promising, but none yet constitutes definitive evidence for average-risk precursor-lesion screening because prospective colonoscopy-verified validation remains limited.

**Figure 2:**
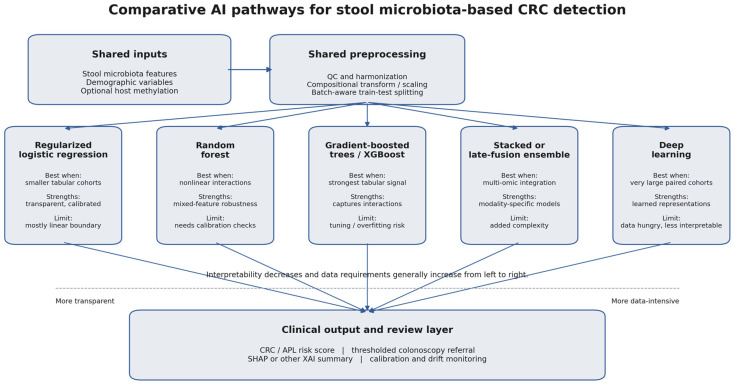
Comparative flow chart of the principal AI model families relevant to stool microbiota-based CRC detection. After shared preprocessing, stool microbiota and demographic inputs can be modeled with regularized linear methods, random forests, gradient-boosted trees, stacked or late-fusion ensembles, or deep learning; the main translational trade-off is between transparency, nonlinear capacity, and data requirements.

For multi-omic stool assays, fusion can be implemented as early fusion (concatenating transformed features), late fusion (combining modality-specific models via ensembling or stacking), or intermediate fusion (learning a joint representation). The choice should be driven by sample size, missingness patterns, and the intended operating point (e.g., fixed specificity). At current sample sizes, late fusion or stacked ensembles often provide the most practical compromise because they allow modality-specific preprocessing, preserve interpretability, and remain usable when one data stream is missing or low quality. Deep learning becomes more compelling when raw sequence embeddings, longitudinal data, or very large, paired cohorts are available. Figure 2 compares the principal AI model families relevant to stool microbiota-based CRC detection and highlights the trade-off between transparency, nonlinear capacity, and data requirements. AI-enabled stool screening therefore offers clear advantages—integration of weak complementary signals, individualized explanations, and flexible operating-point optimization—but also clear liabilities, including batch sensitivity, leakage risk, calibration drift, and greater regulatory complexity than single-analyte tests. Regardless of architecture, developers should prespecify preprocessing, use site-stratified and time-forward validation splits, and report calibration and decision-curve net benefit in addition to discrimination, in line with TRIPOD + AI and related guidance [41,42].

### 3.2. The Generalizability Challenge: Addressing Batch Effects with Advanced Harmonization Frameworks

The single greatest technical obstacle to the widespread clinical deployment of microbiome-based diagnostics is the presence of batch effects. These are systematic, non-biological variations introduced by differences in laboratory protocols, such as sample collection methods, DNA extraction kits, sequencing platforms, and bioinformatic pipelines [29]. Such technical noise can easily obscure or create spurious associations with disease, leading to models that perform well on internal data but fail to generalize to new, external datasets.

Addressing batch effects requires a layered strategy that combines (1) harmonized pre-analytics and laboratory SOPs, (2) batch-aware study design to avoid confounding of site/protocol with case status, and (3) computational harmonization during model development and external validation [29,30]. Several families of batch-correction approaches are used in microbiome studies, including empirical-Bayes methods (e.g., ComBat/ComBat-seq), meta-analysis and mixed-effects frameworks (e.g., MMUPHin), and newer interpretable, compositionality-aware bias-correction models. Each approach carries trade-offs: some assume additive effects on transformed counts, some require sufficient overlap across batches, and supervised approaches must be implemented carefully to avoid label leakage and over-correction of biological signal. DEBIAS-M is one recent interpretable method that estimates protocol-specific, taxon-level bias factors and has been reported to improve cross-study generalization in benchmarking studies [31]. In a consensus framework, DEBIAS-M should be viewed as one option among several; the choice of harmonization method should be prespecified, justified, and evaluated using external-holdout, leave-one-site-out, and time-forward validation designs.

As an illustrative example, investigators can evaluate DEBIAS-M alongside alternative correction strategies (or no correction) within a prespecified analysis plan [31]. Recommended evaluations include leave-one-study-out and external-holdout designs across 16S and shotgun datasets, with metrics spanning discrimination (AUROC/AUPRC), calibration (ECE/Brier), and decision-curve net benefit. Ablation experiments (e.g., protocol-stratified training and partial batch mixing) can help quantify the specific contribution of harmonization to generalizability and identify conditions under which correction helps, fails, or risks removing true biological signal.

### 3.3. Ensuring Clinical Trust: The Role of Explainable AI (XAI)

For any AI-driven diagnostic to achieve clinical adoption, high predictive accuracy is necessary but not sufficient. Clinicians, regulators, and patients require transparency to build trust in the technology. This need has driven the integration of Explainable AI (XAI) techniques into the development process, moving the field toward trust, auditability, and adoption. XAI methods aim to render the decisions of complex black-box models intelligible to human users and are especially important when stool microbiota features are analyzed together with demographic or molecular variables that could otherwise obscure the biological basis of a prediction.

The 2024 study by Novielli et al. provides a clear example of this principle in practice [24]. After training a random forest model to classify CRC based on stool microbiota and demographic data, the investigators applied SHAP, a leading XAI technique, to quantify the contribution of individual taxa and demographic variables to each prediction. The highest-impact drivers included biologically credible CRC-associated taxa such as Fusobacterium, Peptostreptococcus, and Parvimonas, while the explanatory framework also showed how demographic variables can refine baseline risk without replacing disease-associated microbiota signal [24]. Such analyses are important because they distinguish a model that learns biologically plausible CRC patterns from one that merely exploits age, sex, or site-related shortcuts.

Beyond identifying the taxa that drive an individual prediction, XAI in stool-microbiota models should operate at three complementary levels. At the global level, feature-importance summaries and dependence plots can show whether model behavior is dominated by recognized CRC-associated taxa or by unstable technical variables such as collection batch, study site, age, or sex. At the subgroup level, explanation summaries stratified by demographic variables can reveal whether the same taxa contribute similarly across age bands and sex groups, or whether the model is relying too heavily on demographic shortcuts rather than biology. At the local level, patient-specific SHAP outputs can show whether a positive prediction is driven by concordant biological signals or by isolated signals that may reflect inflammation, recent bleeding, or other confounders [24]. For stool microbiota models that incorporate demographic data, this multilevel XAI framework is important because demographic variables often improve calibration but can become dominant predictors if the microbiome signal is weak or unstable. Manuscripts and validation packages should therefore report explanation stability across folds, subgroup-level calibration, and representative examples in which demographic variables appropriately refine rather than replace microbiome-derived biological signal.

## 4. A Development and Validation Playbook for Regulatory and Clinical Success

### 4.1. Pre-Analytical and Laboratory Rigor: The Foundation of a Robust Signal

The successful development of a multi-omic, AI-enabled diagnostic begins long before any algorithm is trained. The quality and consistency of the input data are paramount, as no computational method can rescue a signal corrupted by pre-analytical variability. A rigorous development process must therefore be founded on standardized laboratory protocols. This includes the use of validated stool collection kits with sample stabilizers, a unified protocol for the dual extraction of both host and microbial DNA from the same sample aliquot to ensure direct comparability, and the systematic use of positive (spike-ins) and negative (blanks) controls throughout the workflow. These controls are essential for quantifying and mitigating the effects of low biomass and potential laboratory contamination, ensuring the integrity of the final signal [30].

### 4.2. A Principled AI Development Lifecycle: Adhering to TRIPOD + AI Reporting Standards

The development and validation of the AI model itself must follow a principled, transparent, and reproducible lifecycle. The TRIPOD + AI statement provides the foundational reporting guideline for this process. An update to the original TRIPOD guideline, TRIPOD + AI was specifically designed to harmonize the reporting of clinical prediction models, irrespective of whether they are built using traditional regression or modern machine learning methods [41]. Adherence to its 27-item checklist ensures that key aspects of model development are clearly documented, including the source of data, participant characteristics, handling of predictors and outcomes, sample size justification, methods for model development and internal validation, and a full specification of the final model. This level of transparency is essential for external researchers to critically appraise a model’s performance and risk of bias, and it forms the bedrock of a regulatory submission dossier [43].

### 4.3. Integrating Diagnostic-Accuracy and Bias Appraisal: STARD 2015 and PROBAST

In addition to adhering to TRIPOD + AI for transparent reporting of model development and internal validation, comparative evaluations of diagnostic or screening tests should explicitly follow diagnostic-accuracy reporting standards and include structured risk-of-bias appraisal. The STARD 2015 checklist provides essential items and a recommended flow diagram tailored to diagnostic accuracy studies (index and reference test definitions, timing, and patient flow) and improves completeness and interpretability of test comparisons (Table 2 and Supplementary Figure S1) [44]. Complementing reporting, PROBAST (and the updated PROBAST + AI extension for prediction models using machine-learning/AI) offers a systematic framework to assess risk of bias and applicability across participants, predictors, outcomes, and analysis—a practice that strengthens critical appraisal, meta-analysis, and regulatory submissions when presented alongside TRIPOD + AI items [18,45].

### 4.4. Designing Robust Clinical Trials: Incorporating SPIRIT-AI, CONSORT-AI, and DECIDE-AI Guidelines

Moving a validated model from in silico testing into clinical evaluation requires adherence to a suite of interconnected, AI-specific guidelines. Table 5 summarizes the reporting frameworks most relevant to stool-screening development and implementation, from model development and bias appraisal through prospective trials and early deployment studies. Following this playbook reduces risk in the commercialization process by ensuring that the generated evidence directly answers the questions regulators, payers, and clinical guideline groups are trained to ask. From a clinical and health-system perspective, meaningful impact will likely require advanced precancerous lesion sensitivity that substantially exceeds current stool-based benchmarks (e.g., at least 55–60%) while preserving specificity comparable to established mt-sDNA assays [5,6].

### 4.5. Post-Market Surveillance: Model Governance, Drift Monitoring, and Real-World Evidence

To achieve clinical and payer relevance, future validation must adopt a rigorous prospective design with clearly defined endpoints. The primary endpoint should be advanced precancerous lesion (APL) sensitivity on a per-participant basis, with co-primary specificity. Based on current benchmarks, a clinically meaningful target would be APL sensitivity ≥ 60% with the lower bound of the 95% confidence interval ≥ 50%, while maintaining specificity at approximately 94%, consistent with non-inferiority to Cologuard Plus [5,6]. Key secondary endpoints should include high-grade dysplasia (HGD) sensitivity, stage-stratified CRC sensitivity, predictive values, and colonoscopy yield per 1000 screened. Prespecified subgroup analyses should include adults aged 45–49 (rising incidence but lower absolute prevalence) versus older age groups, as well as sex, race/ethnicity, and recent antibiotic exposure, to characterize transportability and equity of performance. To estimate APL sensitivity with ±5% precision (95% CI), approximately 369 APL cases would be required, corresponding to 3700–5300 participants given an APL prevalence of 7–10% in screening populations. Alternatively, a superiority design to improve from 43% to 60% APL sensitivity at 90% power (alpha = 0.05) would require ~1800–2600 participants per arm [5]. Design considerations include avoiding spectrum bias from case–control enrichment, pre-specifying colonoscopy and histopathology adjudication, and adopting STARD 2015 flow diagrams for transparency [44]. To maximize generalizability, cross-laboratory external validation with harmonized pre-analytics (identical stool kits, dual-extraction SOPs, spike-in controls) should be employed, with correction of protocol-specific biases (e.g., DEBIAS-M) prior to model training [31]. Governance of AI models should include a publicly available Model Card, version control, and preregistration of the locked algorithm per SPIRIT-AI/CONSORT-AI recommendations [46,47,48]. Having defined a pre-market validation strategy consistent with SPIRIT-AI/CONSORT-AI, the post-market lifecycle needed to sustain safety and effectiveness is outlined below.

The lifecycle of an AI diagnostic does not end at regulatory approval. A robust post-market plan is essential. This includes implementing auditable Machine Learning Operations (MLOps) for model governance and change control. Continuous monitoring for performance drift is necessary to detect any degradation in accuracy that may occur as patient populations, clinical practices, or laboratory processes evolve over time. Finally, a framework for leveraging payer-aligned real-world evidence allows ongoing learning, bias audits, model recalibration, and adherence-sensitive value assessment, which is increasingly important for stool-based screening programs [49].

## 5. Implementation and Adoption Considerations

### 5.1. Integration into Existing Screening Infrastructures

The successful translation of a multi-omic stool test requires seamless integration into established screening workflows. Current market leaders have demonstrated that adherence relies heavily on ‘screening enterprises’ that combine diagnostic testing with patient navigation, payer coverage, and electronic medical record (EMR) ordering interfaces. For any new multi-omic entrant, clinical superiority (e.g., improved APL detection) must be paired with operational compatibility—specifically, the ability to utilize existing logistics for at-home collection and to align with HEDIS/Stars quality measures for health systems.

### 5.2. The Evolving Standard of Care

The landscape of noninvasive screening is rapidly shifting. Blood-based assays may be perceived as easier by some patients because they can be bundled with a routine clinic visit and may carry less aversion than stool handling, but they are not inherently more convenient than an at-home stool test because phlebotomy, site access, and scheduling are still required. Current data nevertheless suggest that blood-based assays may lack the sensitivity for advanced precancerous lesions required to support a prevention-first paradigm. In contrast, stool-based approaches, while not universally preferred, currently offer the highest combined sensitivity for cancer and precancer [5]. A next-generation multi-omic test must therefore target a specific clinical utility window: matching the high specificity (~94%) of current benchmarks while significantly exceeding their APL detection rates (currently ~43%) [5,6,7]. In parallel, several stool-based microbiome and RNA initiatives are in development (e.g., Viome and BiotaX), but published prospective, average-risk screening performance data remain limited [11,14].

### 5.3. Intellectual Property and Freedom-to-Operate

The intellectual property landscape for microbiome diagnostics is dense. Broad claims exist regarding the use of specific microbial signatures (e.g., Fusobacterium) and mt-sDNA workflows. A robust translational strategy must move beyond single-biomarker claims and focus on the novel integration layer: proprietary AI fusion algorithms, unique strain-resolved markers, and specific batch-correction methodologies (e.g., DEBIAS-M) [31]. Freedom-to-operate analysis is a critical early-stage requirement to ensure that the resulting diagnostic can be delivered at a scale and cost acceptable to payers.

## 6. Future Directions and Concluding Remarks

### 6.1. The Next Frontier: Strain-Level Resolution, Metabolomics, and Longitudinal Monitoring

The continued advancement of multi-omic screening will likely follow several key research trajectories. One promising avenue is moving beyond species-level taxonomic analysis to strain-level resolution. Different strains of the same bacterial species (e.g., *F. nucleatum*) can have vastly different functional and pathogenic potentials; identifying specific disease-associated clades could significantly increase the precision and specificity of microbial biomarkers [32]. Further integration of other omic layers, such as metabolomics (profiling microbial and host metabolites) and proteomics, may provide a more complete functional picture of the tumor microenvironment and yield complementary diagnostic signals [38]. Finally, the true potential of noninvasive screening may be realized through longitudinal monitoring. Serial stool sampling could enable the development of predictive AI models that detect the dynamic microbial and molecular shifts preceding neoplasia, allowing for risk stratification and intervention before lesions even form.

### 6.2. The Health-Economic Equation: Balancing COGS and Prevention

The shift from targeted PCR to multi-omic profiling (for example, combining targeted host-methylation assays with microbiome sequencing) inherently increases assay complexity and cost-of-goods sold (COGS). While commercial COGS are rarely disclosed in the peer-reviewed literature, order-of-magnitude estimates can help frame feasibility: targeted qPCR panels and targeted methylation assays can be in the tens of dollars per sample for reagents at scale, whereas shotgun metagenomic sequencing (library preparation plus sequencing at a depth suitable for taxonomic classification) is often in the low hundreds of dollars per sample before automation, logistics, and bioinformatics overhead. Developers should therefore define the maximum allowable per-sample COGS early and use decision-analytic models to compare higher upfront testing costs with the prevention dividend of improved APL detection (Table 2), including avoided CRC treatment costs and quality-adjusted life-years [10]. Recent cohort-based modeling that explicitly integrates real-world adherence, lesion detection, and prevention reinforces that the value of stool-based screening depends on program execution and follow-up completion, not assay sensitivity alone [49]. Because the optimal trade-off depends on adherence, referral pathways, and local colonoscopy capacity, the 55–65% APL sensitivity range at approximately 94% specificity is presented here as a pragmatic target window for scenario modeling rather than an empirically proven threshold.

### 6.3. Beyond Colorectal Cancer: Pan-Cancer Applications of Microbial and Multi-Omic Signatures

The technologies and frameworks being pioneered for CRC screening are likely to generalize beyond colorectal neoplasia. AI-assisted microbial or host-microbial profiling is already being explored in oral cancer and broader oncology workflows [50,51], and in refractory inflammatory bowel disease a real-world study reported clinical and inflammatory improvement with an AI-guided multi-omic microbiome modulation strategy [52]. These adjacent use cases do not validate CRC screening directly, but they support the operational feasibility of multi-layer microbial decision support outside a single disease silo and reinforce the broader translational importance of multi-omic modeling.

### 6.4. Ethical and Regulatory Considerations

The translation of AI-enabled multi-omic diagnostics into clinical practice requires adherence to established ethical, regulatory, and safety frameworks. In the United States, the Food and Drug Administration situates AI-enabled diagnostic software within the Software as a Medical Device paradigm and emphasizes Good Machine Learning Practice across the total product life cycle, including dataset curation, performance monitoring, change control, and post-market surveillance [53]. In Europe, AI-based medical or in vitro diagnostic software must now be interpreted at the intersection of the AI Act and the MDR/IVDR frameworks, with emphasis on risk management, data quality, technical documentation, human oversight, and lifecycle accountability [54]. Transparency measures—such as public Model Cards and explainable outputs (e.g., SHAP visualizations)—support clinician oversight and patient trust. Together, these requirements reinforce that regulatory readiness for stool-based AI diagnostics is not only a question of discrimination performance, but also of governance, traceability, and equity.

### 6.5. Limitations

This consensus framework is intentionally forward-looking and therefore has several limitations. First, no large prospective, average-risk screening study has yet evaluated a fully integrated host-methylation plus microbiome stool assay with a locked AI model and colonoscopy as the reference standard; most available multi-omic evidence remains case–control and is focused on CRC rather than APLs, which can inflate apparent sensitivity estimates [36]. Second, microbiome-based signatures show only moderate discriminative performance across cohorts (often AUROC in the ~0.62–0.82 range) and are highly sensitive to batch effects from collection, extraction, sequencing, and bioinformatics pipelines [27,29,30]. For that reason, the microbiome is best viewed as an adjunctive signal to augment host-marker assays rather than as a stand-alone screening solution at present. Although harmonization approaches (e.g., ComBat-seq, MMUPHin, and DEBIAS-M) can improve cross-study transportability, residual technical and population differences may not be fully correctable in all settings [31]. Third, adding metagenomic sequencing (or other high-dimensional profiling) to an at-home workflow increases operational complexity and cost-of-goods; real-world net benefit will depend on adherence, referral pathways, and local colonoscopy capacity. Finally, key confounders—including recent antibiotics, bleeding, inflammation, diet, and comorbidities—can shift microbiome composition and may vary by age and geography; future prospective studies should quantify their impact and test robustness in diverse populations, including adults aged 45–49.

### 6.6. Conclusion: A Consensus Framework for a Clinically Actionable, AI-Powered Diagnostic

The next generation of noninvasive colorectal cancer screening must move beyond incremental improvements in cancer detection and instead focus on closing the persistent gap in advanced precancerous lesion detection. Integrating host DNA methylation markers with gut microbiome features offer a biologically plausible and technologically feasible strategy to capture complementary signals from early tumor biology. However, the clinical credibility of multi-omic stool diagnostics will depend not only on algorithmic innovation but also on rigorous translational methodology. Harmonized sample collection, batch-aware development, leakage-resistant validation, explainable AI outputs, and prospective colonoscopy-verified studies aligned with established reporting standards are essential to ensure reproducibility, regulatory readiness, and clinical trust.

Under these conditions, AI-enabled multi-omic stool screening could shift the emphasis of noninvasive CRC screening from primarily detecting established cancers toward more effective prevention by identifying advanced precursor lesions earlier. The framework presented here therefore outlines a practical pathway for translating emerging microbiome and epigenetic signals into clinically actionable screening tools compatible with real-world screening programs.

## Figures and Tables

**Table 3:** Minimum Translational Checklist.

Domain	Critical Requirement	Rationale for Rigor
Pre-Analytical	Standardized Exclusions: Explicit a priori exclusion of samples with recent antibiotic use (>30 days), overt GI bleeding (>14 days), or recent colonoscopy.	Prevents confounding of microbial and methylation signals by transient physiological states.
	Unified Extraction: Use of validated protocols for dual extraction of host and microbial DNA from the same aliquot.	Ensures direct comparability of multi-omic signals; minimizes batch variation.
AI Validation	Strict Leakage Prevention: Exclusion of all post-referral variables (e.g., hemoglobin immunoassay, pathology logs) from feature sets.	Prevents “hindsight bias” where the model learns proxies for the outcome rather than biological signals.
	Temporal Holdouts: Validation using chronological (time-forward) splits rather than random shuffling.	Simulates prospective clinical deployment and reveals performance drift over time.
Reporting	Guideline Adherence: Full compliance with TRIPOD + AI (development) and STARD 2015 (accuracy) checklists.	Enables transparent critical appraisal by regulators and clinicians.

**Table 4:** Illustrative patient-level SHAP examples: hypothetical values for demonstration only.

Example Patient	True Label	Predicted Prob (Model)	Top Microbiome Contributors (Feature, Direction)—SHAP (Δ Log-Odds)	Top Epigenetic Contributors (Feature, Direction)—SHAP (Δ Log-Odds)	Net Explanation (Short)	Biological Plausibility Note
Patient A (case)	CRC	0.92	Fusobacterium nucleatum—↑ (+1.20); Peptostreptococcus—↑ (+0.45); Parvimonas—↑ (+0.30)	mSEPT9 (high methylation)—↑ (+1.05); SDC2 methylation—↑ (+0.40)	Microbiome and methylation signals are concordant; microbiome shifts + methylated SEPT9 together produce large positive contribution to the call.	Enrichment of Fusobacterium and oral pathobionts is repeatedly associated with CRC.
Patient B (adenoma; APL)	APL	0.68	Fusobacterium—↑ (+0.35); Akkermansia—↓ (protective) (−0.20); Bacteroides fragilis—↑ (+0.25)	SDC2 methylation (moderate)—↑ (+0.50); mSEPT9—low (0.00)	Microbiome provides moderate positive signal; epigenetic SDC2 contributes additional weight yielding an above-threshold call despite low SEPT9.	SDC2 methylation often signals earlier lesions; microbiome shifts can appear in advanced adenomas.
Patient C (false positive/inflammation)	No neoplasia (control)	0.58	High blood-associated taxa signal or recent bleed proxy: Streptococcus—↑ (+0.30); oral microbiome spillover—↑ (+0.25)	mSEPT9—low (0.00); SDC2—low (0.00)	Microbiome-only positive call, no corroborating methylation signal; SHAP shows weaker total contribution vs cases and suggests possible false positive due to inflammation or bleed.	When microbiome signal is driven by bleeding/inflammation, epigenetic markers (host methylation) may help discriminate true neoplasia from confounders.

Notes: The SHAP values shown are hypothetical, illustrative delta log-odds contributions for a single prediction and are not derived from real patients. Positive SHAP pushes probability higher; negative pushes lower. In practice, reports should include patient-level waterfall plots alongside key clinical metadata (e.g., recent antibiotics or bleeding) and should document robustness checks (label permutation, feature ablation, and consistency across folds) to ensure attributions are stable and clinically meaningful. ↑ increase. ↓ decrease.

**Table 5:** Key reporting guidelines for AI in clinical research.

Guideline	Primary Focus	Stage of Research	Key AI-Specific Recommendations (Concise)
TRIPOD + AI	Transparent reporting of model development & validation (regression or ML)	Pre-clinical → internal validation	Full model specification; clearly state data sources and participant flow; describe predictor handling, missingness, model-building, hyperparameter tuning, internal validation (nested CV) and final model specification; provide code/weights where possible [41].
STARD 2015	Diagnostic accuracy study reporting (index vs reference tests)	Comparative diagnostic evaluation/clinical validation	Provide index/reference test definitions, timing, recruitment and participant flow diagrams, blinding/status of readers, handling of indeterminate results, and cross-tabulations (2 × 2). Report test execution and thresholds used; when reporting ML-based index tests, state how thresholds were chosen without leaking test data [44].
PROBAST/PROBAST-AI	Systematic risk-of-bias & applicability appraisal for prediction models	All stages (use for appraisal & reporting)	Perform structured bias assessment across participants, predictors, outcomes, and analysis; for AI models evaluate overfitting, leakage, calibration, temporal/geographic transportability, and transparency of analytical choices. Present PROBAST assessment in the supplement [18,45].
SPIRIT-AI	Trial protocols that include AI interventions	Clinical trial protocol design	Specify algorithm version, intended inputs/outputs, human-AI interaction, monitoring, and plans for data handling/updates; pre-specify primary outcome and evaluation plan.
CONSORT-AI	Reporting of clinical trial results when AI is an intervention	Clinical trial report	Describe AI integration into workflow, versioning, training data provenance, performance error analysis, and state code/model availability and monitoring plans.
DECIDE-AI	Early-stage clinical evaluation & real-world safety	Early clinical evaluation/implementation studies	Assess workflow integration, human factors, clinician decision support, safety monitoring, and iterative refinement; report usability and implementation outcomes.

Taken together, these frameworks are best viewed as a staged evidence stack rather than separate checklists: TRIPOD + AI and PROBAST-AI for model development and bias appraisal, STARD for comparative accuracy studies, SPIRIT-AI and CONSORT-AI for prospective trials, and DECIDE-AI for early deployment studies. For stool-based screening, the most important recurring themes across all six are version locking, transparent participant flow, prespecified thresholds, error analysis, and documentation of how the human-AI team will use the output in practice.

## Data Availability

No new data were created or analyzed in this study. Data sharing is not applicable to this article.

## Supplementary Materials

The following supporting information can be downloaded at: https://www.mdpi.com/article/10.3390/cancers18060909/s1, Table S1. Detailed Pre-analytic Exclusions, Sample-Level Flags, and Mitigation Workflows; Table S2. Comprehensive Feature Handling and Leakage Prevention Strategies; Figure S1. STARD Flow Diagram of Participants; Figure S2. STARD 2015 Checklist; Figure S3: Multi-omic schematic for clinical action; Figure S4. Pre-analytics SOP; Figure S5. Eligibility & Exclusion Criteria; Figure S6. Version-Locked Analysis Plan (PDF); Figure S7. The Multi-Omic “Prevention Prism” Paradigm.
