# Supplementary material for: Defining a Multi-Omic, AI-Enabled Stool Screening Paradigm for Colorectal Cancer: A Consensus Framework for Clinical Translation
Source: Cancers (Basel). 2026 Mar 11;18(6):909. doi: 10.3390/cancers18060909 (PMC13025237; doi:10.3390/cancers18060909)
Supplement: Supplementary file 1 [file cancers-18-00909-s001.zip › cancers-4183684-supplementary.pdf]

Supplementary Table S1. Detailed Pre-analytic Exclusions, Sample-Level Flags, and Mitigation Workflows.

Exclusion/Flag Category	Rationale/Biological Impact	Operational Threshold	Laboratory Action	Mitigation/Re-collection Strategy
Recent systemic antibiotics	Antibiotics alter gut microbiome and can reduce microbial/molecular signal.	Systemic antibiotic use within 30 days prior to sample collection.	Flag sample as exclude for primary analysis; hold sample pending query.	Ask participant to re-collect ≥30 days after completing antibiotics; include as sensitivity analysis if retained. Document antibiotic name/dates.
Overt GI bleeding (visible blood in stool)	Blood can interfere with DNA assays and cause false positives/assay inhibition.	Visible blood reported at collection or obvious blood in returned sample;/or reported GI bleed within 14 days.	Reject for primary analysis; laboratory technician documents sample image/notes.	Request re-collection ≥14 daysafter bleeding resolved. If bleed is ongoing, exclude and advise clinical follow-up.
Colonoscopy / bowel prep proximity	Bowel prep and recent instrumentation dilute/alter stool and cell shedding.	Colonoscopy or bowel prep within 7 days before sample collection.	Flag; exclude from primary cohort unless protocol specifies pre- or post-procedural sampling.	Re-collect ≥7 days after endoscopy and normal bowel function returned. Record procedure date.
Recent rectal exam / hemorrhoidal bleed	Local manipulation may cause blood/altered signal.	Rectal exam, anoscopy, or hemorrhoidal bleeding within 48–72 hours before collection.	Flag; consider exclusion or document and include in sensitivity set.	Re-collect ≥72 hours after local exam/bleeding resolved.
Shipping time / temperature excursions	Temperature/time outside stability envelope degrades nucleic acids and preservatives.	Transit time exceeding validated limit (e.g., >7 days) or recorded temp outside validated range (e.g., >30°C or <0°C) — *use your validated numbers*.	Quarantine sample; inspect preservative integrity; run sample-level QC (internal control).	If preservative/pack damaged or internal control fails, request re-collection ASAP. Implement time-temperature indicators (TTIs) or data-logger monitoring for batch review.
Low biomass / internal control failure	Insufficient host or target DNA → unreliable result (false negatives).	Internal/process control below the lab-validated cutoff (e.g., human DNA Ct above validated limit or signal below LOD).	Flag as low biomass; repeat extraction if reserve aliquot available.	If repeat fails, request re-collection. Consider asking participant about recent laxative/diarrhea/antibiotics that could explain low biomass.
Contamination / batch positive negative control	Laboratory or collection contamination invalidates results.	Any amplification in negative extraction or no-template controls, or unexpected high	Quarantine entire affected batch, repeat extraction/assay;	If contamination confirmed, re-run batch from stored aliquots if available; if not, re-collect affected

		marker in batch blanks.	investigate root cause.	participant samples and document incident.
Inadequate sample volume or preservative damage	Insufficient material to run assay or preservative compromised.	Sample volume < minimum validated (e.g., <X g) or broken/compromised collection tube.	Reject/flag; document photos if available.	Send replacement kit and request re-collection; log impact on yield.
ID/label mismatch or missing metadata	Chain-of-custody and interpretation require correct identifiers and collection metadata.	Missing/illegible barcode, mismatched participant ID, or missing collection date/time.	Hold sample; attempt to resolve with participant/shipper; if unresolved, exclude.	If identity cannot be confirmed, discard and request re-collection; add fail-safe barcode scanning at intake.

This table synthesizes common pre-analytic exclusion criteria and sample-level flags reported across published diagnostic stool-DNA studies, manufacturer/Regulatory SOPs, and laboratory validation protocols. The first column lists the most frequently reported exclusion/flag categories, including recent systemic antibiotics, overt gastrointestinal bleeding, colonoscopy/bowel preparation proximity, and shipping/temperature excursions. The middle column collates representative operational thresholds reported in the literature and industry SOPs (e.g., recommended washout intervals, validated transit limits, and internal control cutoffs), noting that exact numeric cutoffs vary by assay. The final column summarizes typical laboratory actions and mitigation logic, such as intake QC, batch quarantine for contamination events, and predefined re-collection windows. This synthesis highlights common sources of pre-analytic variability that can bias test performance estimates and provides a template for harmonized reporting analogous to STARD/PRISMA expectations.

Supplementary Table S2. Comprehensive Feature Handling and Leakage Prevention Strategies.

Issue / potential leakage source	How it appears in datasets	Mandatory action (model development)	Recommended validation & reporting
**Post-referral clinical variables (label leakage)**	Hemoglobin immunoassay performed after a positive screen; colonoscopy findings, pathology codes, procedure timestamps	**Exclude entirely** from feature set used for model training. Do not use for encoding or imputation.	State explicitly in Methods which variables were excluded (give examples). Provide code listing of excluded fields.
**Temporal leakage (future information)**	Collection/timestamp-based features that encode label timing (e.g., “time to diagnosis”)	Remove or restrict timestamps; use only for *temporal* experiments (train on earlier dates → test on later dates).	Report time-forward split results, calibration drift, and any retraining cadence considered.
**Site / batch confounding**	Site ID, kit lot, lab batch strongly correlated with outcome due to operational differences	Treat as grouping variable: use GroupKFold by site and ensure all samples from a site are in the same fold (do not use site as predictor unless explicitly modeling site effects)	Report per-site performance; perform sensitivity analysis with/without sites dominating signal.

**Preprocessing leakage**	Imputation, scaling, feature selection done before splitting	All preprocessing must be fit inside training folds (pipeline). No global statistics computed on full dataset.	Describe pipeline; report that preprocessing transformers were refit within each fold. Share pipeline code.
**Target-aware encoding / aggregation**	Target encoding across whole dataset or aggregation using outcome labels	Prohibit global target encodings. If encoding uses label proxies, compute only within inner-training folds with appropriate smoothing	Document encoding strategy and demonstrate non-inflation with label permutation tests.
**Duplicate/sample-level leakage**	Multiple samples per participant appearing in train and test	Group by participant so all samples from same person are in one fold	Report grouping method and per-participant split counts.
**Metadata proxies (surrogate labels)**	Features like recent clinical appointment, urgent shipping flag that reflect downstream care	Evaluate and remove if correlated with follow-up; otherwise treat as covariate only in prospective validation	Report correlation of metadata with label and sensitivity analyses excluding these features.
**Calibration/threshold leakage**	Choosing thresholds on full data to report per-1,000 yields	Determine thresholds only from training folds (or separate calibration set) then apply to held-out test data	Report how thresholds were chosen and show held-out application; give confidence intervals from outer folds.
**Undetected contamination / batch artifacts**	Single batch with anomalous high signal driving model	Routine negative/positive control monitoring; permutation tests to spot batch-dominant features	Quarantine affected batches, re-run, or exclude; report control failures and corrective actions.

Prohibits post-referral predictors (e.g., hemoglobin immunoassay results, colonoscopy/pathology-derived variables); all preprocessing, encoding and feature selection are performed **inside** training folds only. Validation uses nested cross-validation with site- and patient-level grouping, plus chronological (time-forward) holdouts to assess temporal drift; routine leakage diagnostics (label permutation, metadata checks) and training-only thresholding are applied.

This table outlines mandatory feature handling protocols designed to prevent data leakage and ensure valid diagnostic performance claims. It categorizes common leakage sources—such as post-referral predictors (e.g., hemoglobin immunoassay results, pathology codes) and target-aware encoding—and specifies mandatory actions for model development, principally that all preprocessing, encoding, and feature selection must occur exclusively within training folds. The table further details recommended validation strategies, including nested cross-validation with site- and patient-level grouping and chronological (time-forward) holdouts to assess temporal drift. Finally, it mandates routine leakage diagnostics, such as label permutation tests and metadata correlation checks, to verify model integrity.

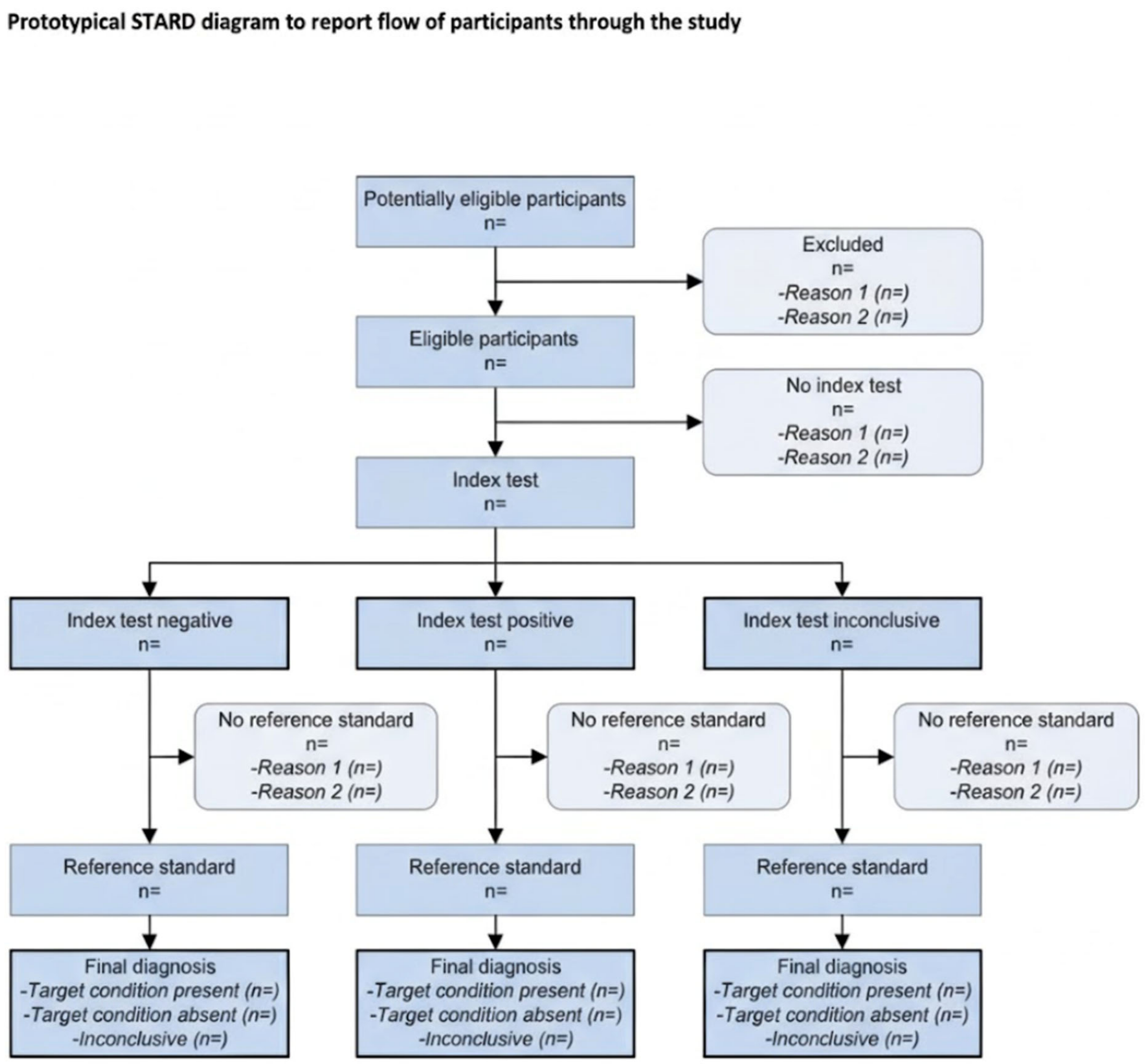

Supplementary Figure S1. STARD Flow Diagram of Participants. The diagram shows the progression of participants through the diagnostic accuracy study, including numbers excluded and the results of the index test and reference standard, in accordance with STARD 2015 reporting guidelines.

STARD 2015 Checklist

STARD 2015 Checklist Items 1 30 (Diagnostic Accuracy Studies)

Instructions: Enter the manuscript page/figure/table where each item is reported. Check NA if not applicable. This template is provided as a companion to the STARD 2015 statement (see EQUATOR Network).

Title/Abstract

1. Identify the study as diagnostic accuracy, including at least one measure of accuracy: ____
2. Structured summary (design, methods, results, conclusions): ____

Introduction

3. Scientific and clinical background, including intended use and clinical role of the index test: ____
4. Study objectives and hypotheses: ____

Methods Participants

5. Study design (prospective/retrospective): ____
6. Eligibility criteria: ____
7. Setting, location, and dates: ____
8. Participant identification/recruitment (consecutive/random/other): ____

Methods Test Methods

9. Index test details (sufficient for replication): ____
10. Reference standard details (sufficient for replication): ____
11. Rationale for choosing the reference standard: ____
12. Definition and rationale for positivity thresholds/cut offs (pre specified vs exploratory) index test: ____
13. Definition and rationale for thresholds reference standard: ____
14. Blinding whether performers/readers of index test had access to clinical info and reference standard: ____
15. Blinding whether assessors of reference standard had access to clinical info and index test: ____
16. Handling of indeterminate results: ____
17. Handling of missing data: ____
18. Analysis methods to estimate/compare accuracy; handling of variability; pre specified subgroups: ____
19. Sample size/how it was determined: ____

Results Participants

20. Participant flow (use STARD diagram; report numbers with reasons): ____
21. Baseline characteristics of participants: ____
22. Disease severity and alternative diagnoses distribution: ____
23. Time interval and any clinical interventions between index test and reference standard: ____

Results Test Results

24. Cross tabulation of index test by reference standard (for target condition): ____
25. Any adverse events from performing index or reference standard: ____

Discussion

26. Study limitations; sources of potential bias; generalizability: ____
27. Implications for practice (including intended use and clinical role of the test): ____

Other Information

28. Registration number and name of registry: ____
29. Where the full study protocol can be accessed: ____
30. Sources of funding and other support; role of funders: ____

Template aligned with STARD 2015 items (EQUATOR Network).

Supplementary Figure S2. STARD 2015 Checklist

This checklist documents item-by-item adherence to STARD 2015 and points to the exact manuscript locations for each element. It enables reviewers to verify transparent reporting of diagnostic accuracy methods and results.

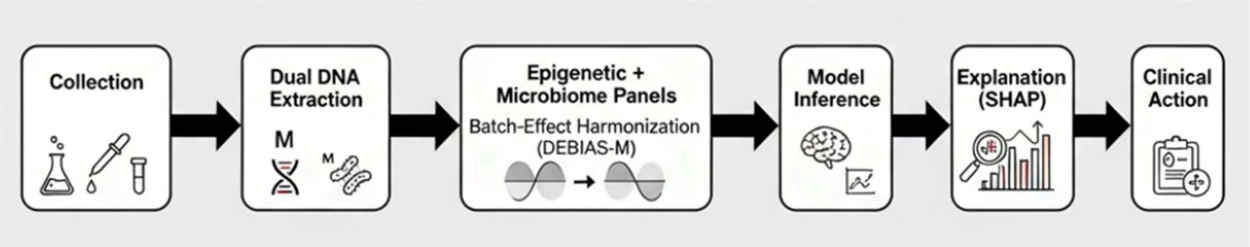

Supplementary Figure S3: Multi-omic schematic for clinical action. The proposed pipeline involves sample collection, dual DNA extraction, and parallel epigenetic/microbiome profiling. Data is harmonized (DEBIAS-M) before being used for model inference. Model predictions are explained using SHAP, with the final output being a clinically actionable insight.

Pre analytic SOPs

Scope
Standard operating procedures for collection, stabilization, logistics, accessioning, dual DNA extraction, library prep, sequencing, and primary QC for a multi omic stool ± blood assay.

Collection & Stabilization
- Approved kits and lot tracking
- Pre collection instructions and eligibility checks -
Stabilization buffer and temperature ranges
- Chain of custody; shipping requirements

Accessioning
- Barcode validation; sample condition scoring
- Rejection criteria (leakage, insufficient volume, broken seal)

Dual DNA Extraction
- Parallel human/microbial DNA protocols; controls (NTC, spike ins, positive controls) - Yield and fragment metrics; contamination alarms

Library Preparation & Sequencing
- Platform/model; cycles; index strategy; pooling rules - Run level
QC; carryover checks

Bioinformatics & Primary QC
- Demultiplexing, trimming, alignment/classification (16S/shotgun), methylation processing - QC thresholds (min reads, host % limits, duplicate %, conversion rate)

Data Acceptance Rules
- Pass/fail gates; repeat/replace logic; documentation templates

Supplementary Figure S4. Pre-analytics SOP

Standardized procedures for collection, stabilization, accessioning, dual DNA extraction, library prep, and QC are outlined to ensure reproducibility. Thresholds and accept/reject rules are specified for auditability.

Eligibility & Exclusion Criteria

Inclusion Criteria (examples; edit to protocol) - Age 45 75, average risk CRC screening

- Asymptomatic; no prior CRC
- Able to provide informed consent and stool sample

Exclusion Criteria (examples; edit to protocol)
- Personal history of CRC, IBD with dysplasia, or hereditary CRC syndromes (e.g., FAP, Lynch) unless predefined

- Colonoscopy within the defined look back window
- Overt gastrointestinal bleeding or alarm symptoms

- Recent antibiotics or bowel prep within exclusion window - Inadequate sample per SOP

Handling of Protocol Deviations

- Document reason; include in CONSORT/STARD flow; prespecified per protocol vs ITT analyses

Supplementary Figure S5. Eligibility & Exclusion Criteria
Full inclusion/exclusion criteria and protocol deviations are reported to define the analyzed population. This supports interpretation of generalizability and potential sources of selection bias.

Version-Locked Analysis Plan (v1.0)

Overview

This version locked analysis plan (VLAP) specifies endpoints, datasets, harmonization, modeling, and evaluation prior to unblinding.

Endpoints

- Primary: CRC detection performance at a pre specified specificity (~94%)
- Key secondary: APL detection; combined (CRC+APL) metrics
- Safety/adverse events related to testing procedures

Datasets

- Derivation, internal validation, and external holdout cohorts (list N, sites, timeframes)
- Reference standards and time intervals

Supplementary Figure S6. Version-Locked Analysis Plan (PDF)

The VLAP prespecifies endpoints, datasets, harmonization settings, modeling, thresholds, and statistical methods prior to unblinding. It records data/code provenance (snapshot date, commit, container, seeds) to ensure reproducibility.

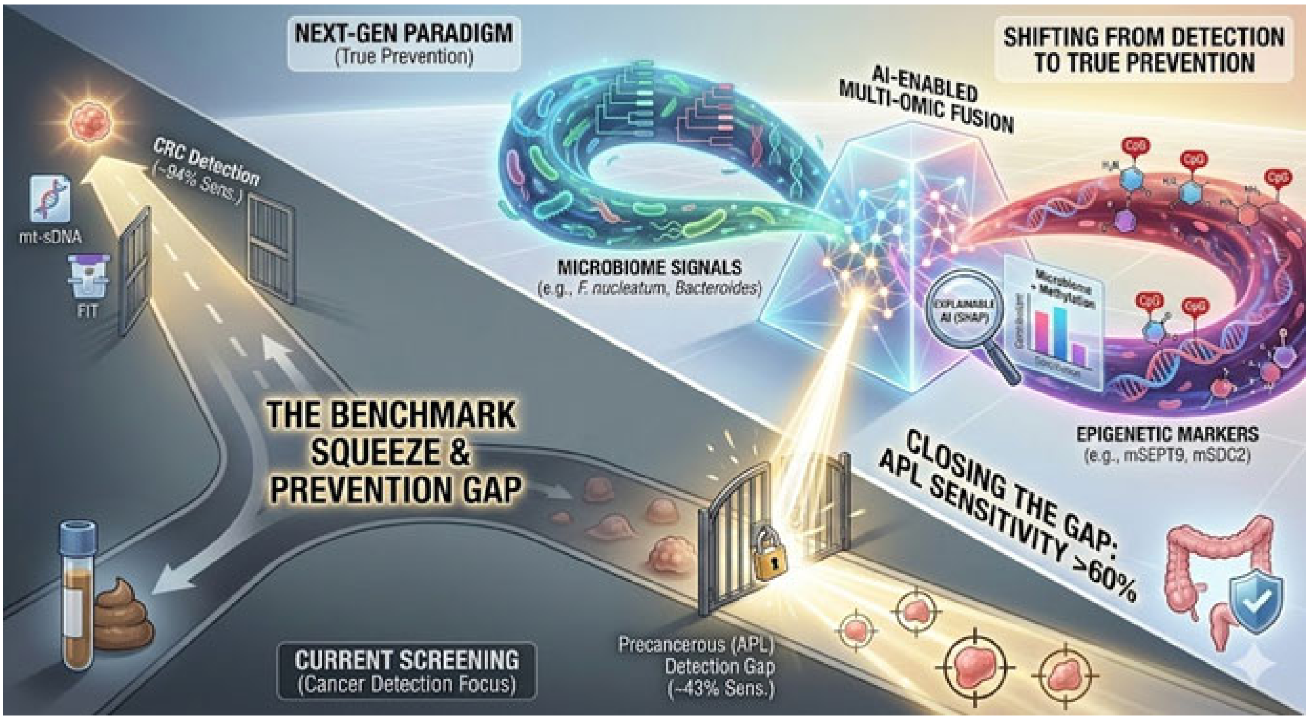

Supplementary Figure S7. The Multi-Omic "Prevention Prism" Paradigm. This conceptual illustration depicts the shift from the current "detection-focused" screening landscape to a "true prevention" model. On the left, existing modalities (FIT, current mt-sDNA) face a "benchmark squeeze," achieving high CRC sensitivity but leaving a critical gap in Advanced Precancerous Lesion (APL) detection (~43%). The central prism represents the proposed AI-enabled fusion engine, which integrates orthogonal signals from host epigenetics (e.g., methylated *SEPT9*, *SDC2*) and the gut microbiome (e.g., *Fusobacterium nucleatum*, *Bacteroides*). By leveraging explainable AI (SHAP) to harmonize these signals, the framework aims to boost APL sensitivity to >60% without sacrificing specificity, effectively unlocking the gate to proactive cancer prevention.
